# Supplementary material for: Chromosome-level genome of the three-spot damselfish, Dascyllus trimaculatus:
Source: G3 (Bethesda). 2023 Mar 11;13(4):jkac339. doi: 10.1093/g3journal/jkac339 (PMC10085752; doi:10.1093/g3journal/jkac339)
Supplement: jkac339_Supplementary_Data [file jkac339_supplementary_data.zip › Supplementary_Figure_1_G3-2022-403614.pdf]

## Supplementary Figure 1.

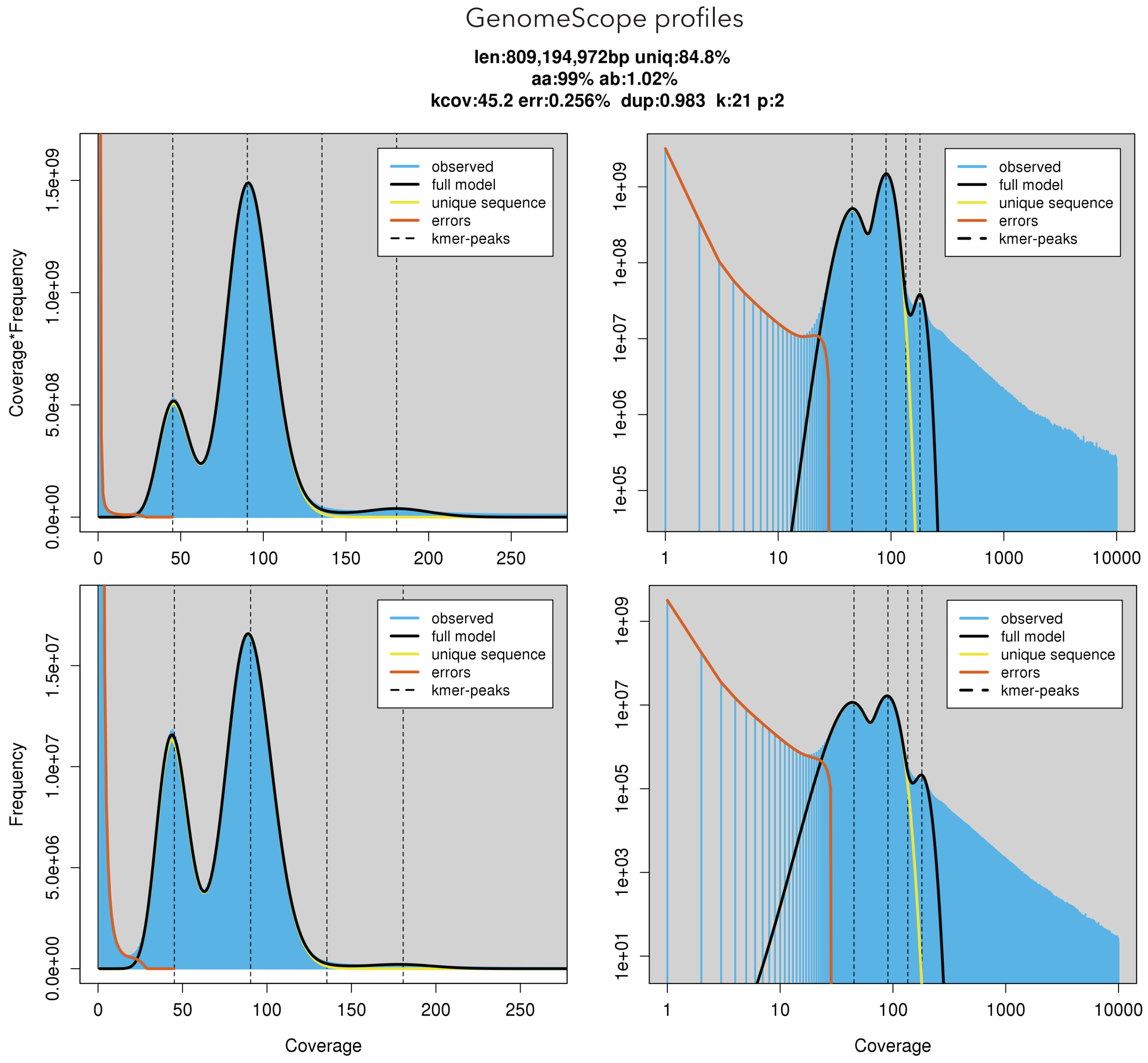


| property | min | max |
|---|---|---|
| Homozygous (aa) | 98.9744% | 98.983% |
| Heterozygous (ab) | 1.01705% | 1.02557% |
| Genome Haploid Length | 808,752,873 bp | 809,194,972 bp |
| Genome Repeat Length | 122,968,760 bp | 123,035,980 bp |
| Genome Unique Length | 685,784,113 bp | 686,158,993 bp |
| Model Fit | 86.9621% | 97.8826% |
| Read Error Rate | 0.256184% | 0.256184% |
