# Supplementary material for: Chromosome-level genome of the three-spot damselfish, Dascyllus trimaculatus:
Source: G3 (Bethesda). 2023 Mar 11;13(4):jkac339. doi: 10.1093/g3journal/jkac339 (PMC10085752; doi:10.1093/g3journal/jkac339)
Supplement: jkac339_Supplementary_Data [file jkac339_supplementary_data.zip › Supplementary_Table_1_G3-2022-403614.docx]

**Table S1.**

Sequencing information of data used to assemble the genome of the three-spot damselfish, *Dascyllus trimaculatus*, Bioproject: PRJNA828170 Biosample: SAMN27642109; Genome accession: JAMOIN000000000 for isolate Kuro_0920G. All data is available at https://www.ncbi.nlm.nih.gov/sra/PRJNA828170.

| Purpose | Data Type | Linker Sequence | Sequencing Chemistry | Sequencing Institution | Number of bases | Coverage | GenBank accession |
| --- | --- | --- | --- | --- | --- | --- | --- |
| High accuracy short reads | NEB Next Ultra II WGS |  | HiSeq 4000 150PE | UC Davis | 94383867000 | 103.6x | SRX17663068 |
| Proximity ligation | Chicago - DpnII | GATCGATC | HiSeq 4000 150PE | Fulgent Genetics | 19712640600 | 21.6x | SRX17663071 |
|  | Chicago - MluCl | AATTAATT |  |  | 22305540600 | 24.5x | SRX17663072 |
|  | Chicago - Fatl | CATGCATG |  |  | 20104123200 | 22.1x | SRX17663073 |
|  | HiC - DpnII | GATCGATC |  |  | 32486064300 | 35.7x | SRX17663069 |
|  | HiC - MluCl | AATTAATT |  |  | 45729378900 | 50.2x | SRX17663070 |
| Long-reads for scaffolding | ONT-MinION |  | SQK-LSK109/FlowCell 9.4.1 | UCSC | 4789005362 | 5.3x | SRX17742644 |
|  |  |  |  |  | 15220684630 | 16.7x | SRX17742645 |
